# A Still Greater Effort Required

**Published:** 1921-07-23

**Authors:** 


### THE FINANCING OF THE LONDON HOSPITALS.

### A Still Greater Effort Required.

A Special Meeting of the President and General Council of King Edward's Hospital Fund for London was held at St. James's Palace on Wednesday morning, July 13. In the unavoidable absence of His Royal Highness the Prince of Wales, President of the Fund, the Earl of Donoughmore, K.P., presided.

Lord Somerleyton (Hon. Secretary) read the following letter from H.R.H. the Prince of Wales:

"St. James's Palace, S.W.,
"July 12, 1921.

"Dear Lord Somerleyton,—

"It is a matter of great regret to me that I cannot be present at the meeting of the General Council to consider the recommendations of Lord Cave's Committee. The meeting had to be held this week as a matter of urgency, and I am unfortunately unable to attend. I am glad to see that Lord Cave's Committee reported so strongly in favour of the voluntary hospital system, and expressed their belief that the hospitals, if only they could be tided over the next two years by emergency grants from the Government, would be able to recover from the effects of the war and to re-establish their finances. If the Government cannot do as much as Lord Cave's Committee think will be required, a still greater effort is required of the hospitals and the central funds, backed, I hope, by the public, to carry out the suggestions in Lord Cave's report; and thus to avert the injury to the welfare of the sick and the enormous expense to the State which the Report rightly says would follow if the voluntary hospital system fell to the ground.—Believe me, yours very truly,

"Edward P."

Lord Donoughmore said he was sure they all greatly regretted His Royal Highness's absence, and sincerely hoped it would be only a short time before he was able to return to his normal work, which was very great.

The appointment of the following new members of the General Council and Committees was notified: General Council: Viscount Hambleden; Finance Committee: Mr. M. C. Norman; Policy Committee: Sir Basil E. Mayhew.

New Duties for the King's Fund.

The Chairman of the King's Fund Policy Committee (Lord Stuart of Wortley) presented a Preliminary Report of that Committee on the final Report of Lord Cave's Committee. The Report includes a brief summary of the principal recommendations of Lord Cave's Committee affecting the King's Fund. The new duties which would fall on it if the recommendations of Lord Cave's Committee are adopted would be—(*a*) To nominate one member of a Hospitals Commission of twelve—[this has already been done.—Ed. The Hospital]—the chief function of which would be to administer the temporary Government assistance; (*b*) to report to the Hospitals Commission on the circumstances of hospitals in the Metropolitan police district applying for emergency assistance, and as to the conditions that should accompany grants; (*c*) to perform the other functions of a Voluntary Hospitals Committee for the Metropolitan police district.

The Committee considers it important that the hospitals should accept the expression of opinion of Lord Cave's Committee in regard to the development of systematic weekly contributions from wage-earners as the basis of immediate practical experiment, since they would thus show that they are working along lines indicated by that Committee, with every intention of satisfying the authorities that they are in earnest in carrying out the proposed conditions of emergency grants. It therefore at once called a conference of the twelve teaching hospitals for June 14, and followed this up by a circular to all the hospitals and an invitation to a larger conference on July 6, the object being to stimulate and assist the various institutions to decide which method they preferred, and to take immediate steps, separately or in co-operation, to prepare practical schemes. It also acted at once on the recommendation of Lord Cave's Committee that the peculiar difficulties of organising wage-earners' contributions in London should form the subject of early discussion between the King's Fund and the Hospital Saturday Fund. A first meeting of representatives of the two Funds was held on June 16.

Bridging the Gap.

The Committee states it does not follow that the various methods of developing systematic weekly contributions from wage-earners with or without voluntary insurance are necessarily suitable or adequate for all hospitals, or that they are the only methods by which hospitals can carry out the conditions that would attach to emergency grants; but they appear to be those that should first be investigated. Amongst other methods, Lord Cave's Committee lays great stress on economies of expenditure that may be required as a condition of the grant. They refer in this connection to the investigation of comparative cost by the King's Fund, and make a number of suggestions for the reduction of expenditure. The Policy Committee suggests that the General Council should do what it can to shorten the interval that must in any case elapse before emergency assistance can actually reach the hospitals of London which are in danger of "immediate disaster." It also thinks it desirable that, in order to emphasise the urgent need for the hospitals to take immediate action to re-establish their finances within the period of probation suggested by Lord Cave's Committee, the Fund should publicly state, at the earliest possible moment, that the efforts made by the various hospitals in this direction will be taken into account by the Fund at its ordinary distribution. If this is approved the Policy Committee proposes to take into consideration, under the powers already delegated to it by the General Council, the question on what general basis this

(*Continued on page* 282.)

### The Financing of the London Hospitals (*continued from page* 280.)

policy could best be carried out, and to make a recommendation to the President and General Council later on.

Danger of Delay Emphasised.

Lord Stuart of Wortley pointed out that Lord Cave's Committee said that a million pounds was wanted to give the hospitals time to reorganise their finances, and in view of the proved deficits in the London area the £500,000 suggested by the Government was clearly, upon the face of of it, not enough. Urgency and prompt action were, therefore, all the more required. On behalf of the King's Fund Policy Committee he moved the following resolution:

That the General Council of King Edward's Hospital Fund

(*a*) thanks Lord Cave's Committee for its sympathetic and clear-sighted survey of the problem, and in particular for the emphasis laid by the Report upon the value of individual effort and initiative in hospitals, and for the fruitful suggestions of methods by which hospitals could restore their finance;

(*b*) welcomes the recognition by Lord Cave's Committee that none of these new methods will immediately produce a large revenue, and that the next two years must be regarded as an emergency period during which a Government grant on a sufficiently substantial scale will be necessary, subject to such conditions and administered by such machinery as would secure the maintenance of the voluntary system;

(*c*) considers that the sum of £1,000,000 recommended by Lord Cave's Committee for the year 1921 would probably be required for this purpose, since the evidence in the possession of the King's Fund goes to show that the deficits at the London hospitals alone are likely to amount to about half this sum;

(*d*) desires to emphasise the fact stated by Lord Cave's Committee, that, solely as a consequence of the war, many of the hospitals are at present carried on at a large weekly deficit, and that the position of hospitals throughout the country is thereby imperilled;

(*e*) urges the consequent danger from a delay of even a few weeks, and the importance of prompt action to carry out the recommendations of Lord Cave's Committee, in order to avoid immediate disaster to the hospitals and the sick poor, and the enormous expense to the Exchequer which would follow if the voluntary system were to collapse and hospitals had to be provided by public funds.

The motion, seconded by the President of the Royal College of Physicians (Sir Norman Moore), and supported by Lord Mersey, was, after discussion, put and carried, and it was resolved that copies be forwarded to the Prime Minister, the Chancellor of the Exchequer, and the Minister of Health, and to the members of Lord Cave's Committee.

On the motion of Lord Stuart of Wortley, seconded by Sir Cooper Perry, it was resolved—

That the King's Fund Policy Committee be and they hereby are authorised to inform the hospitals that the Fund will, at the ordinary distribution for the current year, take into account the action adopted by the various hospitals to carry out the recommendation of Lord Cave's Committee that they should take all steps that are open to them to re-establish their financial position within the next two years.

